# Vitamin D disrupts NS1-TUFM interaction to suppress pathogenic mitophagy in RSV-induced mitochondrial injury of bronchial epithelial cells

**DOI:** 10.71150/jm.2508009

**Published:** 2026-01-31

**Authors:** Li Peng, Yao Liu, Xiaofang Ding, Tuhong Yang, Lili Zhong, Fangcai Li

**Affiliations:** 1 Hunan Provincial Key Laboratory of Pediatric Respirology, Pediatric Medical Center, Hunan Provincial People's Hospital and First Affiliated Hospital of Hunan Normal University, Changsha 410005, P. R. China; 2 Hunan Provincial Center for Disease Control and Prevention, Changsha 410005, P. R. China

**Keywords:** Vitamin D, RSV NS1, TUFM, mitophagy, RSV bronchiolitis

## Abstract

This study aims to examine the mechanism by which vitamin D mitigates bronchiolitis caused by respiratory syncytial virus (RSV) through the regulation of RSV nonstructural protein 1 (NS1)-TUFM-mediated mitophagy in bronchial epithelial cells. Clinical serum and PBMC samples from RSV-infected children and healthy controls were analyzed for vitamin D, mitochondrial DNA, mitophagy markers (LC3, ATG5, VDAC1, TOMM20, and COXIV), TUFM, and inflammatory cytokines (IL-6, IL-8, and TNF-α). In vitro, human bronchial epithelial cells Beas-2B were transfected with RSV-NS1 plasmid and TUFM silencing or overexpression constructs. Vitamin D (0.1–10 μM) was administered to evaluate mitophagy inhibition using Western blot, immunofluorescence, and JC-1 staining. NS1-TUFM interaction was confirmed by co-immunoprecipitation. RSV-positive patients exhibited reduced serum vitamin D, elevated TUFM and mitophagy markers, impaired mitochondrial mass, and increased inflammation. Vitamin D inversely correlated with LC3 and TUFM. RSV-NS1 overexpression induced mitochondrial translocation of NS1, TUFM-dependent mitophagy activation, and mitochondrial dysfunction (JC-1 depolarization). Vitamin D (10 μM) suppressed mitophagy by redistributing NS1 to the cytosol and reducing mitochondrial TUFM. TUFM overexpression abolished the protective effects of vitamin D on mitophagy and inflammation. In conclusion, vitamin D inhibits mitophagy in bronchial epithelial cells infected with RSV by disrupting NS1-TUFM interaction, suggesting that the vitamin D-TUFM axis may serve as a potential therapeutic target.

## Introduction

Viral bronchiolitis is the leading cause of infant hospitalization in high-income countries, with respiratory syncytial virus (RSV) responsible for 60–80% of cases (Dalziel et al., 2022). Although RSV shares transmission routes with other respiratory viruses, it exhibits unique pathogenic mechanisms (Jartti et al., 2019), including the manipulation of host cell autophagy pathways (Chen et al., 2024b; Li et al., 2018). A key viral factor in this process is the non-structural protein 1 (NS1). This protein localizes to mitochondria and is a potent suppressor of interferon signaling. Furthermore, NS1 delays apoptosis in infected cells; its early expression activates the anti-apoptotic phosphoinositide-dependent protein kinase (PDK)/protein kinase B (AKT)/glycogen synthase kinase (GSK) pathway, thereby prolonging the viral replication cycle and increasing progeny yield (Bitko et al., 2007).

The specific form of autophagy manipulated by RSV is mitophagy, the selective degradation of mitochondria (Lu et al., 2023). This process is triggered through a direct interaction between NS1 and the mitochondrial Tu translation elongation factor (TUFM). The NS1-TUFM interaction facilitates the mitochondrial localization of NS1 and initiates TUFM-dependent mitophagy, which has been directly linked to lung injury (Cheng et al., 2023). Importantly, pharmacological inhibition of autophagy attenuates RSV-induced inflammation and damage, underscoring the pathogenic role of this pathway and its therapeutic potential (Huang et al., 2024). However, the specific contribution of the NS1-TUFM axis to the pathology of RSV bronchiolitis requires systematic investigation.

Concurrently, compelling clinical evidence links vitamin D deficiency to increased susceptibility and severity of RSV infection. Children hospitalized with RSV bronchiolitis exhibit significantly lower serum vitamin D levels (Alakaş et al., 2021), with severity inversely correlating with vitamin D status (Gao et al., 2023). Vitamin D exerts immunomodulatory effects via nuclear receptor signaling, suppressing pro-inflammatory cytokines and regulating antimicrobial peptides (Hamza et al., 2023; White, 2022). Importantly, experimental studies reveal that vitamin D3 modulates mitophagy in lung epithelium, reducing mitochondrial fission and repressing TNF-α-induced ROS production (Chen et al., 2022) . In models of RSV-associated asthma, vitamin D treatment ameliorates lung injury by suppressing autophagy (Huang et al., 2022), suggesting potential interaction with mitochondrial mass control pathways. Despite these connections, the specific impact of vitamin D on the RSV NS1-driven, TUFM-dependent mitophagy pathway remains unexplored.

To circumvent biosafety constraints of live RSV, we employed NS1 plasmid transfection models combined with clinical analysis. We hypothesize that vitamin D attenuates bronchiolitis by suppressing NS1-TUFM-mediated mitophagy, thereby restoring mitochondrial integrity and reducing inflammation. This work aims to elucidate a novel protective mechanism of vitamin D against RSV bronchiolitis, disruption of the NS1-TUFM axis, and inhibition of pathogenic mitophagy, identifying both vitamin D and the TUFM pathway as potential therapeutic targets and revealing a new regulatory link between nutritional status and mitochondrial mass control during viral infection.

## Materials and Methods

### Clinical sample collection

13 RSV-positive children hospitalized with bronchiolitis (RSV group) and 10 age-matched healthy controls (Normal group) were enrolled. Their clinical characteristics are presented in Table 1. Inclusion criteria: (1) Confirmed RSV infection via nasopharyngeal swab testing (RSV antigen or nucleic acid detection); (2) Complete clinical records; (3) Written informed consent from guardians. Exclusion criteria: (1) Co-infection with other pathogens; (2) Congenital heart disease, chronic lung disease, immunodeficiency, prematurity (< 36 weeks), or systemic steroid treatment within 2 weeks prior to admission. Following the collection of fasting venous blood, a portion of the samples underwent centrifugation (3,000 × g, 15 min) to isolate serum for the detection of inflammatory cytokines, vitamin D, and mitochondrial DNA release levels. A separate subset of samples yielded peripheral blood mononuclear cells (PBMCs) for assessment of mitochondrial autophagy-related protein expression. PBMCs were isolated from buffy coat samples via centrifugation over Ficoll-Paque and cultured in medium containing 10% FBS, supplemented with recombinant human IL-2 (rhlL-2; 50 U/ml), rhlL-7 (10 ng/ml), and rhIL-15 (5 ng/ml).

### Enzyme-linked immunosorbent assay (ELISA)

Patient serum and supernatants from Beas-2B cultures were collected. Serum vitamin D level was quantified utilizing an ELISA kit (EU2541; FineTest, China). Additionally, IL-6, IL-8, and TNF-α levels were quantified by ELISA kits sourced from Proteintech (China), with catalogue numbers KE00139, KE00275, and KE00154, respectively. The operational procedures were strictly followed according to the experimental manual provided by the manufacturer.

### Quantitative reverse transcription polymerase chain reaction (qRT-PCR)

Total RNA was extracted with TRIzol (15596026, Thermo Fisher), reverse-transcribed (CW2569, CWBIO), and amplified using SYBR Green (CW2601, CWBIO). The primer sequences are listed in Table 2. The relative gene expression levels were analyzed by calculating the comparative cycle threshold values (2^-ΔΔCt^) with *GAPDH* normalization.

### Cell culture and treatment

Human bronchial epithelial cells (Beas-2B; AW-CNH004, Abiowell, China) were cultured in DMEM (iCell-0001, iCell) supplemented with 10% fetal bovine serum (FBS) and 1% penicillin-streptomycin at 37°C under 5% CO_2_. Beas-2B cells were chosen because they are a well-established model for human bronchial epithelium and are highly relevant to RSV infection. Cells (2.5 × 10^6^/well in 6-well plates) were transfected with 1 μg RSV-NS1 plasmid (oe-RSV-NS1; pCIneo-RSV-NS1 DNA) or control plasmid (oe-NC; empty pCIneo vector). The NS1 expression vector carries a GFP fluorescent tag. Following transfection, NS1-positive cells were isolated by flow cytometry and verified using fluorescence. Additionally, TUFM siRNA (si-TUFM) or overexpression plasmid (oe-TUFM) was developed and synthesized by Honorgene (China). Transfection was performed using Lipofectamine 2000 (11668019; Invitrogen, USA) per the manufacturer’s protocol. Cells were harvested 24 h post-transfection. Vitamin D (HY-15398, MedChemExpress) was dissolved in ethanol and diluted in culture medium. Cells were subjected to treatment with varying concentrations of vitamin D (0.1, 1, and 10 μM) for 24 h. To stimulate mitophagy, a concentration of 20 μM carbonyl cyanide m-chlorophenyl hydrazone (CCCP; HY-100941, MedChemExpress) was employed as a positive control for 6 h.

### Western blot analysis

Proteins were extracted from Beas-2B cells using RIPA buffer. Mitochondrial and cytoplasmic fractions were isolated utilizing the mitochondria isolation kit (89874, Thermo Fisher). Equal quantities of protein from each sample were subjected to separation via SDS-PAGE and subsequently transferred to nitrocellulose membranes. Following a 1-h blocking period with a specific buffer, the membranes were incubated overnight at 4°C with primary antibodies, as detailed in Table 3. In the analysis of proteins within the cytoplasm and total cell lysate, GAPDH serves as an internal reference. Conversely, when assessing mitochondrial proteins, voltage-dependent anion channel 1 (VDAC1) is employed as the internal reference. This was followed by incubation with diluted horseradish peroxidase (HRP)-conjugated Affinipure goat anti-rabbit IgG (AWS0002, Abiowell) and HRP-conjugated Affinipure goat anti-mouse IgG (AWS0001, Abiowell). The resultant protein bands were visualized using the Chemiluminescence Imaging System (ChemiScope6100, CLINX, China). Protein expression levels were quantified using ImageJ software.

### Immunofluorescence staining

Beas-2B cells were seeded on coverslips in 24-well plates and treated as indicated. After treatment, cells were fixed with 4% paraformaldehyde (AWI0056a, Abiowell) for 30 min at room temperature and permeabilized with 0.3% Triton X-100 (AWI0603a, Abiowell) for 30 min at 37°C. After three washes with PBS, cells were incubated with 3% hydrogen peroxide for 15 min at room temperature in the dark to quench endogenous peroxidase activity. Following three PBS washes, cells were blocked with 5% BSA (AWI0120a, Abiowell) for 60 min at 37°C. For dual labeling of LC3 and translocase of outer mitochondrial membrane 20 (TOMM20), a sequential staining protocol was employed using the Tyramide Signal Amplification (TSA) kit (AWI0693a, Abiowell). Primary antibodies against LC3 (1:200; 14600-1-AP, Proteintech) or TOMM20 (1:200; 11802-1-AP, Proteintech) were applied overnight at 4°C, followed by incubation at 37°C for 2 h. After washing with PBS, horseradish peroxidase (HRP)-conjugated secondary antibodies (AWS0002, Abiowell) were applied for 30 min at 37°C. TSA fluorescence dyes (620 nm for LC3 and 520 nm for TOMM20) were then applied for 10 min at room temperature. After the first target staining, antibody elution was performed to remove the primary-secondary complex. Cells were incubated with antibody elution buffer (AWI0707, Abiowell) at 37°C for 10 min twice, followed by PBS washes. The same procedure was repeated for the second target. Finally, nuclei were counterstained with DAPI (5 μg/ml; AWC0293a, Abiowell) for 10 min at 37°C. Coverslips were mounted with anti-fade buffer (AWT0124a, Abiowell) and imaged using a fluorescence microscope (BA210T, Motic) at 400× magnification. Quantitative analysis of immunofluorescence images was performed using ImageJ software. At least three independent replicate samples were analyzed per experimental group, with no fewer than ten randomly selected fields of view per sample, totaling over 300 cells analyzed. LC3-positive puncta were defined as discrete foci larger than 5 pixels in size with fluorescence intensity exceeding twice the background threshold. Mitochondrial membrane potential in JC-1-stained cells was expressed as the ratio of the average red fluorescence intensity to the average green fluorescence intensity per cell. All data are presented as Mean ± standard error of the mean. Intergroup comparisons were performed using one-way analysis of variance (ANOVA) followed by Tukey's post hoc test, with *P* < 0.05 considered statistically significant.

### Mitochondrial membrane potential (MMP) assay

MMP was determined via JC-1 staining (C2006, Beyotime), where a decrease in red/green fluorescence ratio (aggregate/monomer forms) indicated depolarization. The specific experimental procedure follows the manufacturer's instructions.

### Co-Immunoprecipitation (Co-IP)

Lysates from oe-RSV-NS1-transfected cells were incubated with anti-TUFM antibody. Complexes were pulled down with Protein A/G beads and analyzed by Western blot.

### Statistical analysis

Data are expressed as Mean ± standard deviation (n = 10 normal subjects and 13 RSV-positive patients in clinical sample testing, n = 3 independent experiments in cell experiments). Comparisons between groups were analyzed using Student’s *t*-test and one-way ANOVA, followed by post-hoc Dunnett’s test (GraphPad Prism 9.0). Pearson correlation analysis was employed to examine the associations between vitamin D levels and inflammatory cytokines (IL-6, IL-8, and TNF-α), mitochondrial DNA, LC3, and TUFM levels. *P* < 0.05 was considered statistically significant.

## Results

### RSV-infected patients exhibit significant mitochondrial damage, accompanied by upregulation of TUFM and reduced vitamin D levels

**Fig. 1.**
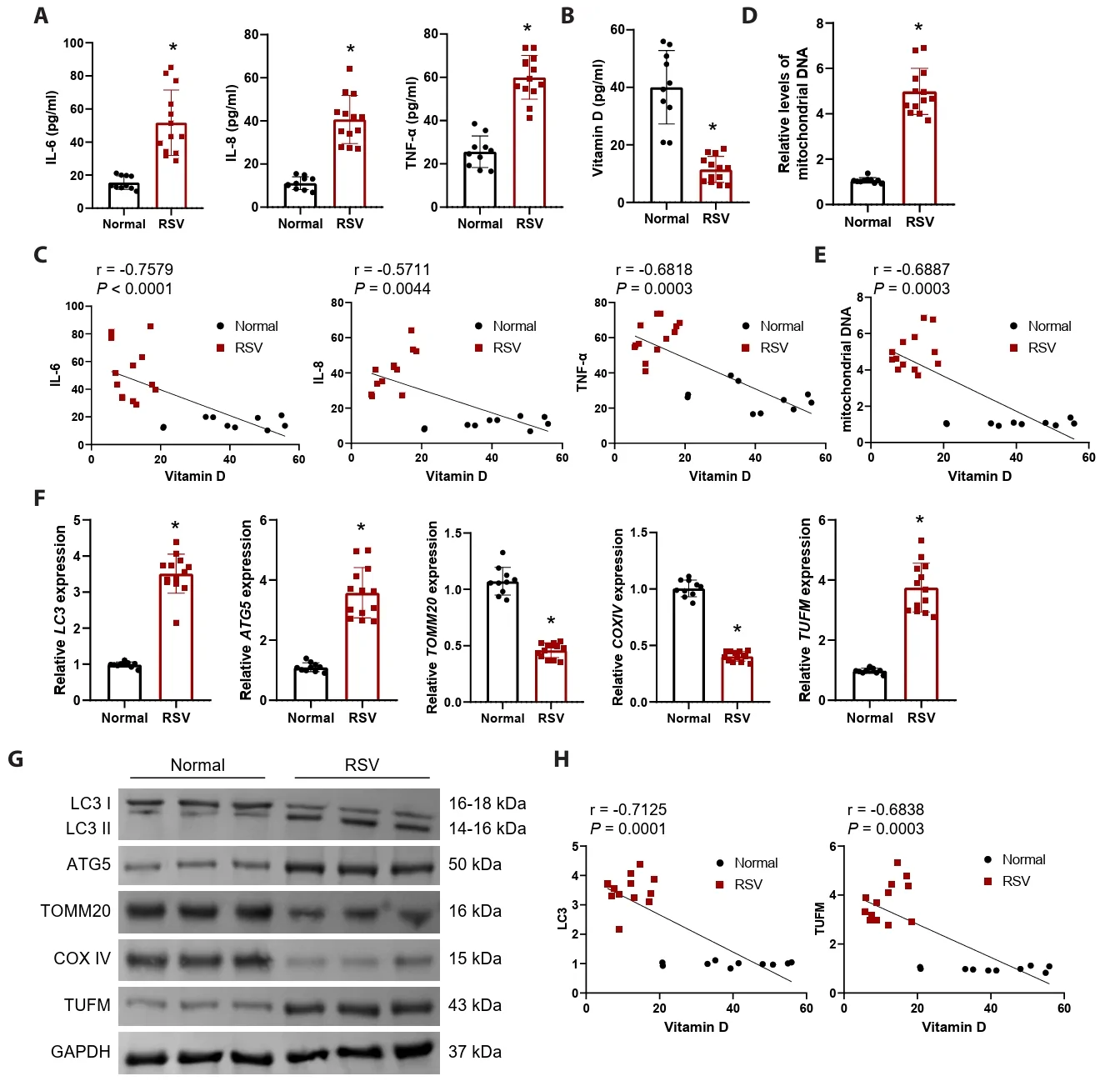
RSV-infected patients exhibit significant mitochondrial damage, accompanied by upregulation of TUFM and reduced vitamin D levels. Serum samples were obtained from 13 RSV-positive children hospitalized with bronchiolitis (RSV group) and 10 age-matched healthy controls (Normal group). (A) Serum IL-6, IL-8, and TNF-α levels were measured to assess systemic inflammatory response. (B) Detection of vitamin D levels in the serum. (C) Pearson correlation analysis of IL-6, IL-8, and TNF-α levels with vitamin D levels, respectively. (D) Mitochondrial DNA release was quantified by measuring the ratio of mitochondrial *COX2* DNA to nuclear *GAPDH* DNA using qRT-PCR. (E) Pearson correlation analysis of mitochondrial DNA release levels with vitamin D levels. (F–G) The mRNA and protein levels of mitophagy markers (*LC3*, *TUFM*, *ATG5*, *TOMM20*, and *COXIV*) in PBMCs were detected by qRT-PCR and western blot. (H) Pearson correlation analysis of *LC3* and *TUFM* levels with vitamin D levels, respectively. n = 10 normal subjects and n = 13 RSV-positive patients. The unpaired *t*-test was employed for comparisons between the two groups. ^*^P < 0.05 vs. Normal group.

In this study, we enrolled 13 hospitalized patients diagnosed with RSV infection and 10 healthy control participants. Despite comparable demographics and basic hematological parameters, RSV-infected patients exhibited significant elevations in respiratory rate, C-reactive protein, and lactate dehydrogenase (Table 1), indicating a state of systemic inflammation and cellular injury. This was further corroborated by significantly elevated serum inflammatory cytokines (IL-6, IL-8, and TNF-α) (Fig. 1A). Numerous studies have established a significant relationship between vitamin D levels and RSV infection (Gao et al., 2023; Huang et al., 2022). In alignment with these findings, our results demonstrated a significant reduction in serum vitamin D levels in the context of RSV infection (Fig. 1B), with a negative correlation to IL-6, IL-8, and TNF-α (Fig. 1C). To assess the involvement of mitochondrial damage in the course of RSV infection, we measured mitochondrial DNA levels. Results demonstrated a significant elevation in mitochondrial DNA levels in patients infected with RSV (Fig. 1D), suggesting that mitochondrial damage occurs within cells following RSV infection, leading to the release of DNA into the serum. Furthermore, serum vitamin D levels exhibited a significant negative correlation with the level of mitochondrial DNA released (Fig. 1E).

Given the pivotal role of mitophagy in the context of viral infections (Li et al., 2022), we assessed the expression of autophagy markers LC3 and autophagy related 5 (ATG5) in PBMCs, alongside mitochondrial proteins TOMM20 and cytochrome c oxidase IV (COXIV). Our findings revealed a significant upregulation of mRNA and protein levels for *LC3* and *ATG5*, while *TOMM20* and *COXIV* exhibited a notable downregulation (Fig. 1F and 1G). This suggests that mitophagy is substantially activated during RSV infection, resulting in diminished mitochondrial mass. The RSV-NS1 protein is recognized as a crucial element for effective viral replication (Ottenio de Lourenço et al., 2022). Recent investigations have indicated that RSV-NS1 interacts with TUFM to promote TUFM-dependent mitophagy (Cheng et al., 2023). Consequently, we further analyzed *TUFM* mRNA and protein levels in PBMCs, which were found to be significantly elevated (Figs. 1F, 1G, and S1A), indicating that RSV infection triggers TUFM-dependent mitophagy. Pearson correlation analysis revealed a significant negative correlation between serum vitamin D levels and both LC3 and TUFM mRNA levels (Fig. 1H). These observations indicate that mitophagy is enhanced in the context of RSV infection, potentially linked to the upregulation of TUFM and the reduction of vitamin D levels.

### RSV-NS1 protein activates mitophagy in Beas-2B cells

**Fig. 2.**
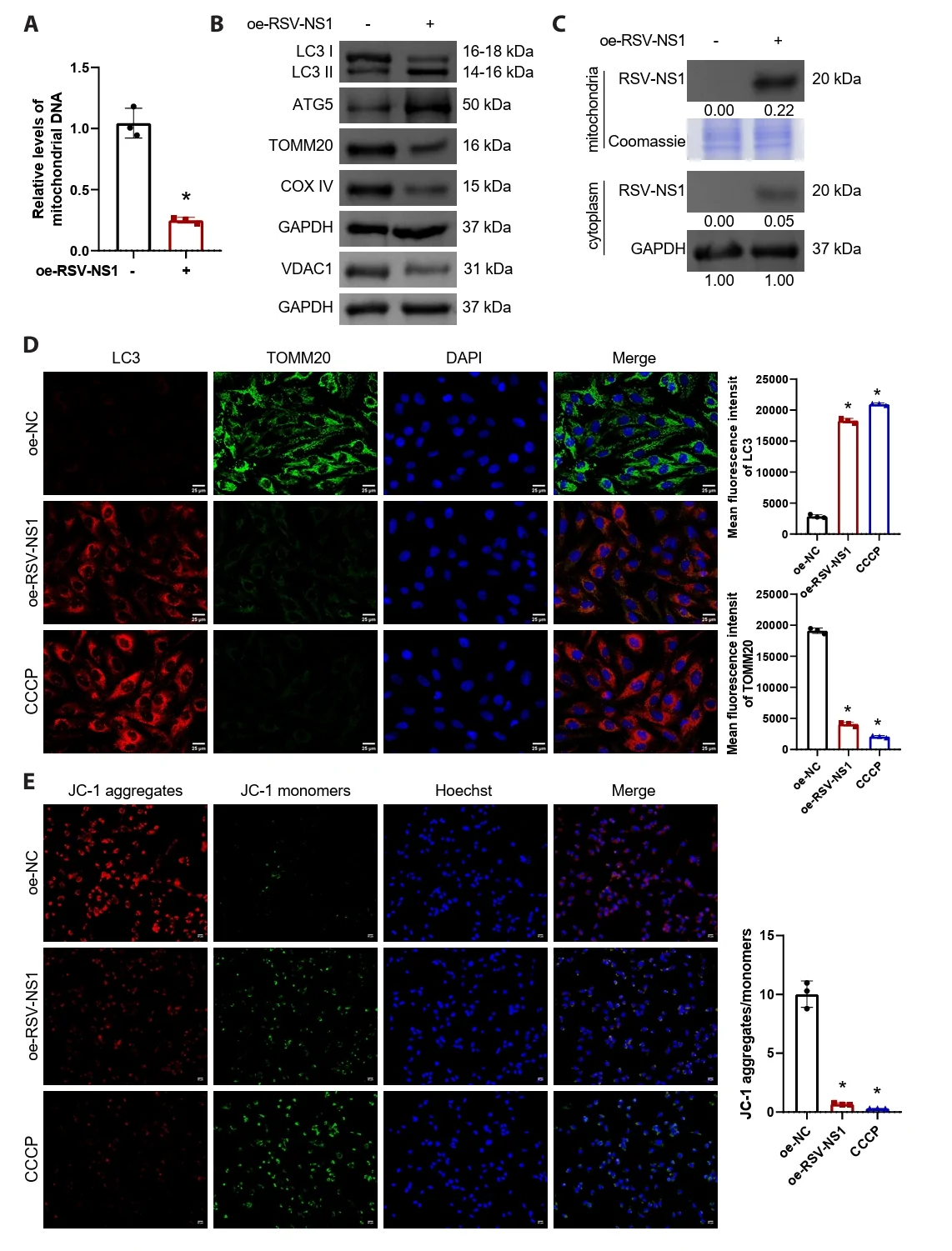
RSV-NS1 protein activates mitophagy in Beas-2B cells. (A) Mitochondrial DNA levels in Beas-2B cells were quantified by measuring the ratio of mitochondrial *COX2* DNA to nuclear *GAPDH* DNA using qRT-PCR. The observed effects on mitochondrial DNA release are attributed to the specific overexpression of the NS1 protein. The unpaired *t*-test was employed for comparisons between the two groups. (B) Western blot detection of LC3, ATG5, VDAC1, TOMM20, and COXIV in Beas-2B cells. The unpaired *t*-test was employed for comparisons between the two groups. (C) Western blot detection of RSV-NS1 protein levels in mitochondria and cytoplasm. Coomassie blue staining is depicted as an internal reference for mitochondrial proteins, while GAPDH was used as an internal reference for cytoplasmic proteins. The data were normalized with the GAPDH as a loading control. (D) Immunofluorescence colocalization analysis of LC3 and TOMM20 to assess mitophagy. Scale bar = 25 μm. (E) Assessment of mitochondrial membrane potential using immunofluorescence methods for JC-1 staining. Scale bar = 50 μm. n = 3. Intergroup comparisons were performed using one-way ANOVA followed by Tukey's post hoc test. ^*^P < 0.05 vs. oe-NC group.

To investigate the regulatory influence of the RSV-NS1 protein on mitophagy, we conducted transfections of either the RSV-NS1 plasmid or a control plasmid into normal human bronchial epithelial cells, specifically Beas-2B. Subsequently, NS1-positive cells were sorted using flow cytometry for subsequent experiments and validated using GFP fluorescence (Fig. S2A and S2B). The findings revealed a significant reduction in intracellular mitochondrial DNA levels (Fig. 2A), suggesting that RSV-NS1 overexpression induces mitochondrial damage and releases DNA into the extracellular space. Furthermore, overexpression of RSV-NS1 markedly increased autophagy markers LC3-II/I and ATG5, while concurrently downregulating mitochondrial proteins voltage-dependent anion channel 1 (VDAC1), TOMM20 and COXIV (Figs. 2B and S1B). The lipidated form of LC3B, referred to as LC3-II, serves as a recognized marker for autophagosomes. Established research demonstrates that TOMM20 and VDAC1 are degraded together during mitophagy (Fan et al., 2021). This observation suggests that, in alignment with RSV infection (Cheng et al., 2023), the expression of RSV-NS1 protein markedly stimulates mitophagy. Prior studies have demonstrated that RSV-NS1 localizes to mitochondria through its interaction with the mitochondrial protein TUFM (Cheng et al., 2023). Consequently, we isolated mitochondrial and cytoplasmic proteins from Beas-2B cells that stably overexpress RSV-NS1 to examine their cellular distribution. Figure S3 demonstrates the successful separation of cytoplasmic and mitochondrial components. Our analysis indicated that the RSV-NS1 protein predominantly accumulates in the mitochondria, with a minor presence in the cytoplasm (Fig. 2C), corroborating previous findings (Ottenio de Lourenço et al., 2022). CCCP, a well-known mitochondrial oxidative phosphorylation uncoupler, is frequently employed to depolarize mitochondria and initiate mitophagy (Kwon et al., 2017), and thus served as a positive control. We investigated the role of RSV-NS1 in regulating mitophagy and mitochondrial function through a combined immunofluorescence approach. Mitophagy flux was assessed by quantifying the co-localization of LC3 puncta with TOMM20-labeled mitochondria. Image analysis confirmed that RSV-NS1 expression significantly enhanced this co-localization, to an extent similar to the CCCP positive control (Fig. 2D). Furthermore, MMP was evaluated using the JC-1 probe. Consistent with enhanced mitophagy, RSV-NS1 expression resulted in a significant decrease in the red/green fluorescence intensity ratio, indicating MMP dissipation, as also observed in CCCP-treated cells (Fig. 2E). These data lead us to conclude that the RSV-NS1 protein localizes to mitochondria to activate mitophagy, which in turn impairs mitochondrial function.

### RSV-NS1 protein activates mitophagy through TUFM in Beas-2B cells

**Fig. 3.**
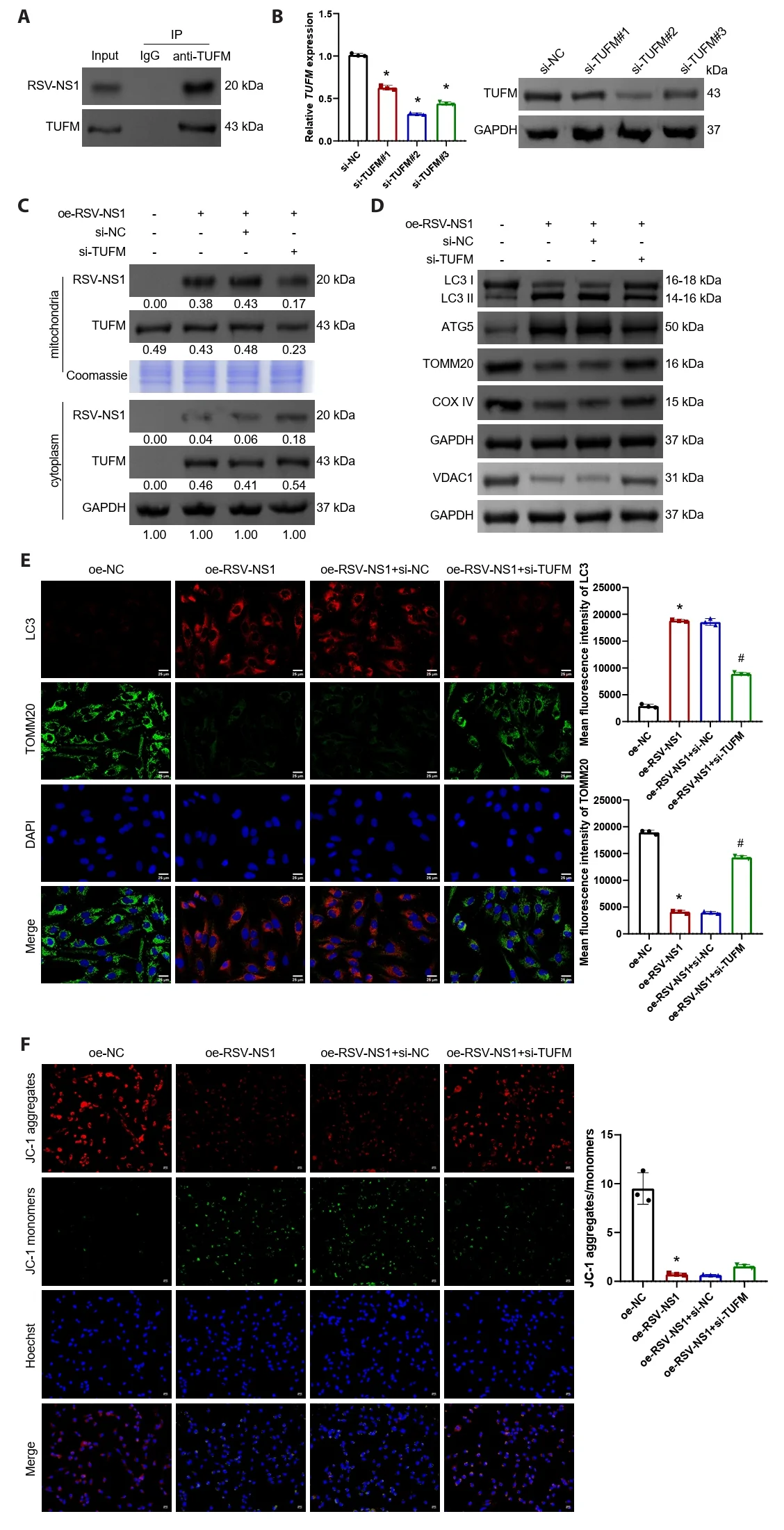
RSV-NS1 protein activates mitophagy through TUFM in Beas-2B cells. (A) The interaction between RSV-NS1 and TUFM was assessed using co-immunoprecipitation techniques. (B) TUFM silencing plasmids (#1–#3) or control plasmids were transfected into Beas-2B cells. The mRNA and protein levels of *TUFM* were detected using qRT-PCR and Western blot. ^*^P < 0.05 vs. si-NC group. (C) Western blot detection of RSV-NS1 and TUFM protein levels in the mitochondria and cytoplasm fraction. Coomassie blue staining is depicted as an internal reference for mitochondrial proteins, while GAPDH was used as an internal reference for cytoplasmic proteins. The data were normalized with the GAPDH as a loading control. (D) Western blot detection of LC3, ATG5, VDAC1, TOMM20, and COXIV in Beas-2B cells. (E) Immunofluorescence colocalization analysis of LC3 and TOMM20 to assess mitophagy. Scale bar = 25 μm. (F) Assessment of mitochondrial membrane potential using immunofluorescence methods for JC-1 staining. Scale bar = 50 μm. n = 3. Intergroup comparisons were performed using one-way ANOVA followed by Tukey's post hoc test. ^*^P < 0.05 vs. oe-NC group; ^#^*P* < 0.05 vs. oe-RSV-NS1+si-NC group.

In Beas-2B cells transfected with the RSV-NS1 plasmid, the interaction between RSV-NS1 and TUFM was confirmed (Fig. 3A). Following this, Beas-2B cells were transfected with TUFM silencing plasmids, and the transfection efficiency was assessed using qRT-PCR and Western blot analysis (Figs. 3B and S1C). The silencing efficiency was found to be highest at target #2, which was subsequently utilized for further experiments. The findings indicated that silencing TUFM led to a reduction in its protein levels within the mitochondria, while the levels in the cytoplasm remained relatively stable (Fig. 3C). Moreover, TUFM silencing facilitated the translocation of RSV-NS1 protein from the mitochondria to the cytoplasm without affecting its overall protein levels (Fig. 3C). Western blot analysis revealed that TUFM silencing restored the levels of LC3-II/I and ATG5, which had been upregulated by RSV-NS1 protein, while simultaneously upregulating the expression of VDAC1, TOMM20, and COXIV (Figs. 3D and S1D). Immunofluorescence staining for TOMM20 and LC3, in conjunction with JC-1 staining, further corroborated that TUFM silencing inhibits the mitophagy induced by RSV-NS1 expression and partially ameliorates mitochondrial dysfunction (Fig. 3E and 3F). These results indicate that the RSV-NS1 protein interacts with the mitochondrial protein TUFM, thereby activating mitophagy and compromising mitochondrial function.

### Vitamin D inhibits mitophagy activated by RSV-NS1 protein

**Fig. 4.**
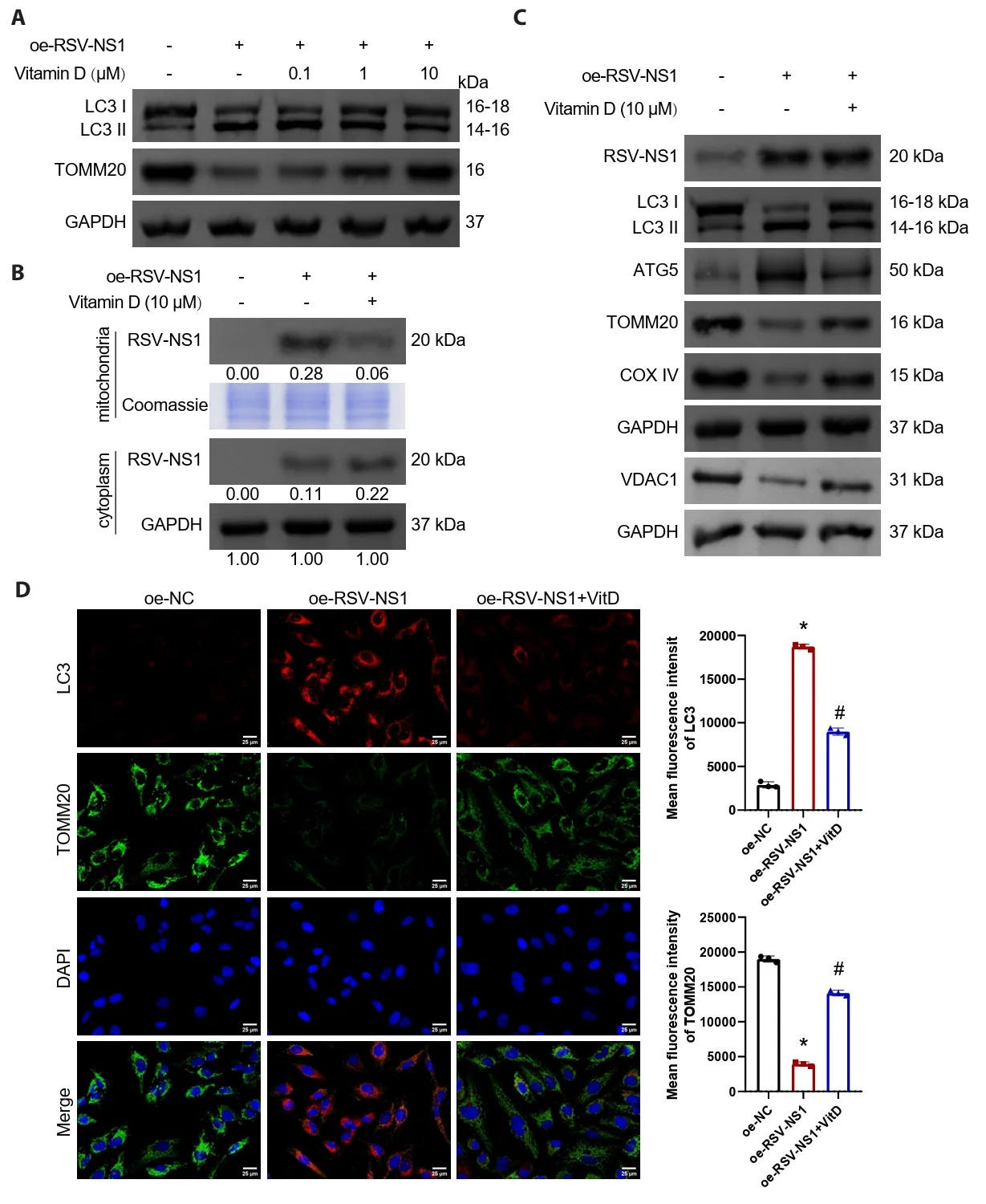
Vitamin D inhibits mitophagy activated by RSV-NS1 protein. Beas-2B cells that express RSV-NS1 were subjected to treatment with varying concentrations (0.1, 1, 10 μM) of vitamin D. (A) Western blot detection of LC3 and TOMM20 in Beas-2B cells. Subsequently, Beas-2B cells overexpressing RSV-NS1 were subjected to a 10 μM vitamin D intervention. (B) Western blot detection of RSV-NS1 protein levels in mitochondria and cytoplasm. Coomassie blue staining is depicted as an internal reference for mitochondrial proteins, while GAPDH was used as an internal reference for cytoplasmic proteins. The data were normalized with the GAPDH as a loading control. (C) Western blot detection of RSV-NS1, LC3, VDAC1, ATG5, TOMM20, and COXIV in Beas-2B cells. (D) Immunofluorescence colocalization analysis of LC3 and TOMM20 to assess mitophagy. Scale bar = 25 μm. n = 3. Intergroup comparisons were performed using one-way ANOVA followed by Tukey's post hoc test. ^*^P < 0.05 vs. oe-NC group; ^#^*P* < 0.05 vs. oe-RSV-NS1 group.

Numerous studies have indicated that vitamin D possesses the ability to inhibit damage associated with mitophagy (Chen et al., 2022; Lai et al., 2022; Lee et al., 2020). In our investigation, we treated Beas-2B cells expressing the RSV-NS1 protein with varying concentrations of vitamin D. The findings demonstrated that vitamin D effectively mitigated mitophagy activated by the RSV-NS1 protein and increased mitochondrial mass in a concentration-dependent manner (Figs. 4A and S1E). Notably, treatment with 10 μM of vitamin D produced the most pronounced therapeutic effect, leading to its selection for subsequent experiments. We assessed the levels of RSV-NS1 protein in both mitochondrial and cytoplasmic fractions, in addition to the levels of RSV-NS1 protein in whole cell lysates. Our analysis revealed that vitamin D facilitated the translocation of RSV-NS1 protein from the mitochondria to the cytoplasm (Fig. 4B), while not influencing the overall levels of RSV-NS1 protein (Figs. 4C and S1F). Furthermore, the observed reductions in LC3-II/I and ATG5 levels, alongside the upregulation of VDAC1, TOMM20, and COXIV, provided additional evidence supporting the therapeutic efficacy of vitamin D in modulating mitophagy and increasing mitochondrial mass (Figs. 4C, 4D, and S1F). Additional experiments confirmed that vitamin D’s inhibition of RSV-NS1 overexpression-induced mitophagy was comparable to that of the autophagy inhibitor Bafilomycin A1 (Fig. S4). Collectively, these results suggest that vitamin D can inhibit mitophagy activated by the RSV-NS1 protein in normal human bronchial epithelial cells.

### Vitamin D alleviates RSV-NS1 protein-induced mitophagy and inflammatory response via TUFM

**Fig. 5.**
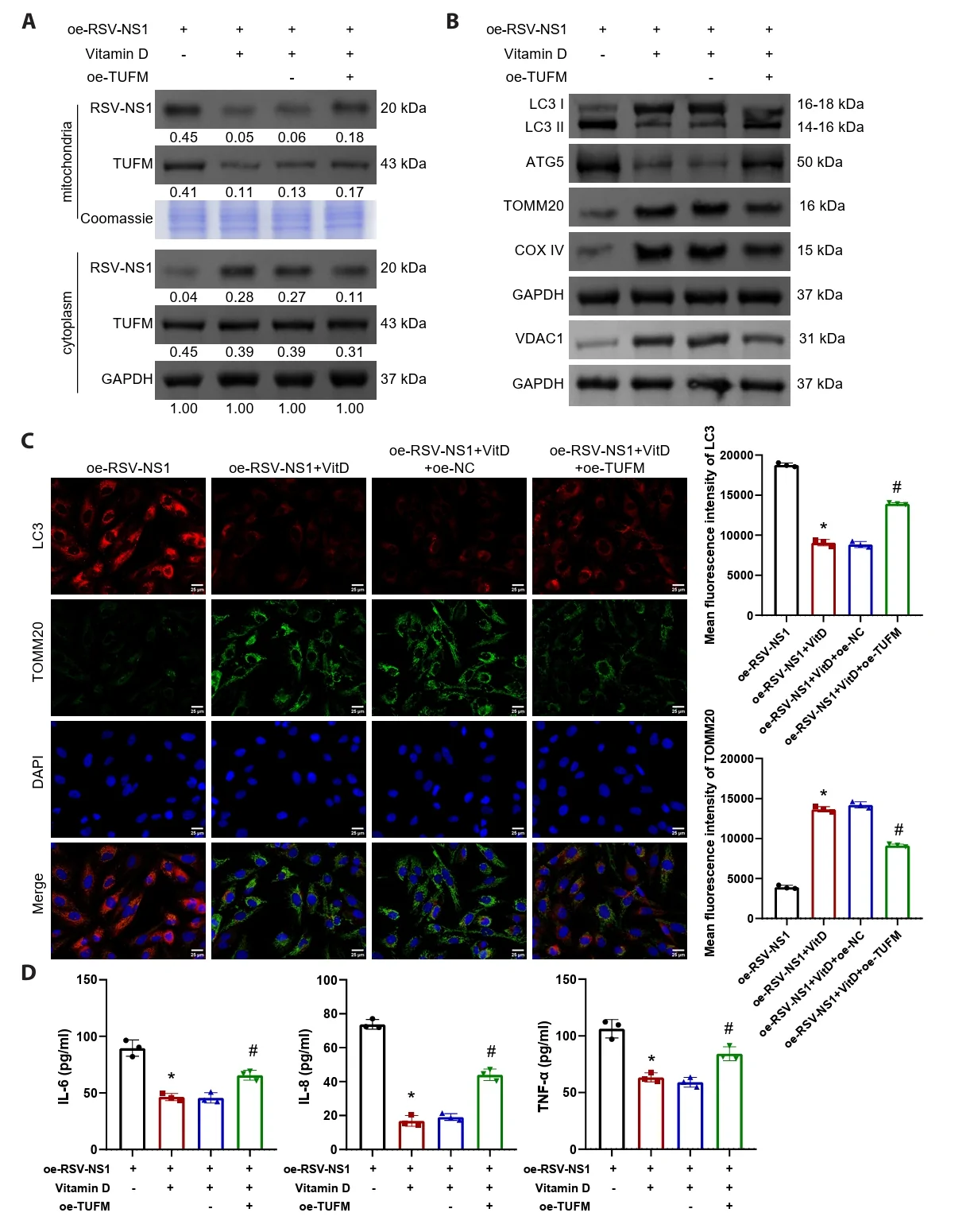
Vitamin D alleviates RSV-NS1 protein-induced mitophagy and inflammatory response via TUFM. (A) Western blot detection of RSV-NS1 and TUFM protein levels in mitochondria and cytoplasm. Coomassie blue staining is depicted as an internal reference for mitochondrial proteins, while GAPDH was used as an internal reference for cytoplasmic proteins. The data were normalized with the GAPDH as a loading control. (B) Western blot detection of LC3, ATG5, VDAC1, TOMM20, and COXIV in Beas-2B cells. (C) Immunofluorescence colocalization analysis of LC3 and TOMM20 to assess mitophagy. Scale bar = 25 μm. (D) The concentrations of IL-6, IL-8, and TNF-α in the supernatant of Beas-2B cells were quantified to evaluate the inflammatory response. n = 3. Intergroup comparisons were performed using one-way ANOVA followed by Tukey’s post hoc test. ^*^P < 0.05 vs. oe-RSV-NS1 group; ^#^*P* < 0.05 vs. oe-RSV-NS1+VitD+oe-NC group.

The findings presented indicate a negative correlation between serum vitamin D levels and TUFM levels in patients infected with RSV (Fig. 1E). To further elucidate the involvement of TUFM in the therapeutic effects of vitamin D, Beas-2B cells expressing the RSV-NS1 protein were treated with vitamin D while simultaneously co-transfected with a TUFM overexpression plasmid. The results demonstrated that vitamin D facilitated the translocation of RSV-NS1 protein from the mitochondria to the cytoplasm, concurrently decreasing TUFM protein levels within the mitochondria (Fig. 5A). Notably, the overexpression of TUFM partially counteracted this effect. Western blot and immunofluorescence analyses collectively indicated that TUFM overexpression significantly intensified the mitophagy and impaired mitochondrial mass, which were alleviated by vitamin D treatment (Figs. 5B, 5C, and S1G). Furthermore, vitamin D markedly reduced the inflammatory response elicited by the RSV-NS1 protein, as evidenced by the increased levels of inflammatory cytokines IL-6, IL-8, and TNF-α (Fig. 5D). This therapeutic effect was similarly diminished following TUFM overexpression. These findings indicate that vitamin D downregulates the mitochondrial protein TUFM, thereby mitigating the mitophagy and inflammatory responses induced by the RSV-NS1 protein.

## Discussion

This study demonstrates that vitamin D alleviates TUFM-mediated mitophagy initiated through the RSV-NS1 protein. By combining clinical data from children with mechanistic studies in NS1-transfected airway epithelium, we observed that serum vitamin D levels were significantly decreased in RSV-infected patients, while TUFM expression, mitophagy markers, and pro-inflammatory cytokines were concurrently elevated. This aligns with cohort studies linking hypovitaminosis D to severe RSV disease (Alakaş et al., 2021; Gao et al., 2023). The inverse relationship between vitamin D levels and mitophagy markers in patients suggests that vitamin D may confer protection through modulation of mitochondrial turnover pathways. These clinical correlations provide a foundation for our mechanistic investigations, though future longitudinal studies should establish whether vitamin D deficiency precedes infection or results from it.

Among the RSV proteins, NS1 was chosen as the primary focus because it is a well-established master virulence factor that localizes to mitochondria and potently suppresses interferon signaling (Boyapalle et al., 2012; Cheng et al., 2023; Van Royen et al., 2022). The mitochondrial protein TUFM was selected based on compelling prior evidence and our own confirmation of its physical interaction with NS1, positioning it as a critical node linking viral infection to mitophagy and immune suppression (Chen et al., 2024a; Lei et al., 2012; Zhang et al., 2025). While we acknowledge that other viral or mitochondrial proteins may participate in the host response to RSV, the NS1-TUFM complex represents a uniquely well-defined and mechanistically rich interface that is strongly supported by existing literature. Our study was designed to deeply characterize this specific, high-priority pathway. Future investigations will undoubtedly explore the potential involvement of other candidates.

At the molecular level, we establish that RSV-NS1 protein alone suffices to induce pathogenic mitophagy through direct interaction with TUFM. The RSV NS protein, as the earliest and most highly expressed viral protein, is known to inhibit the interferon pathway. Interestingly, its activity requires the assembly of a large NS degradosome complex on mitochondria, which comprises the NS1 protein and several unknown host factors (Goswami et al., 2013). This finding resonates with recent work by Cheng et al. (2023) showing NS1 hijacks mitophagy via TUFM in HEp-2 cells. This consistency across different cell types (HEp-2 and our Beas-2B model) suggests the NS1-TUFM axis may be a fundamental mechanism rather than a cell type-specific phenomenon. The redistribution of NS1 from mitochondria to cytosol upon TUFM silencing confirms TUFM’s essential role as a mitochondrial anchor for NS1 (Chen et al., 2024a). This relocalization correlates with restored mitochondrial integrity, supporting the concept that TUFM-dependent mitochondrial targeting of NS1 drives epithelial damage. Notably, our observation that TUFM knockdown reduces mitochondrial NS1 without altering total NS1 levels suggests a specific role in subcellular trafficking rather than protein degradation.

Mitophagy is recognized as a pivotal mechanism regulating innate antiviral responses. Numerous viruses, including measles virus and Epstein-Barr virus, have been reported to hijack mitochondrial autophagy to suppress host innate immune pathogenesis (Vilmen et al., 2021; Xia et al., 2014). Our study confirms that RSV-NS1 overexpression induces mitophagy in Beas-2B cells, while Baf A1 pretreatment alleviates this effect.

In our study, vitamin D exerts protective effects by disrupting the NS1-TUFM-mitophagy axis through two complementary mechanisms: reduction of mitochondrial TUFM pools and displacement of NS1 from mitochondria. This dual action distinguishes it from canonical mitophagy inhibitors like 3-MA, which broadly target autophagy initiation (Zhang et al., 2021). Our data align with reports that vitamin D reduces TNF-α-induced mitophagy in lung epithelium (Chen et al., 2022) but reveal a novel viral-specific mechanism through TUFM downregulation. Similarly, vitamin D effectively alleviates oxidative stress, mitophagy, and inflammation in mouse aortas induced by particulate matter (PM_2.5_) (Lai et al., 2022). Notably, the concentration-dependent inhibition of mitophagy by vitamin D, with maximal efficacy at 10 μM. Crucially, TUFM overexpression completely reversed vitamin D’s benefits on mitophagy markers, MMP, and inflammation, establishing TUFM as the primary effector mediating vitamin D’s action in this pathway. Contrastingly, other studies report that vitamin D expression enhances mitophagy in cardiomyocytes from D-galactose-induced aging rats (Shahidi et al., 2023) and hyperthyroid rats (Shokri et al., 2024). This discrepancy suggests that vitamin D's ultimate effects may depend on specific pathophysiological contexts, tissue types, and the initial triggers of mitochondrial damage.

In our research design, we adopted complementary experimental models to bridge clinical phenomena with molecular mechanisms. At the clinical level, we selected PBMCs as a readily accessible biological sample to assess the systemic immunometabolic state triggered by RSV infection. Although not the primary viral target, PBMCs act as circulating "immune sentinels" that reflect systemic stress responses to infection, including mitochondrial alterations (Zhou et al., 2020). In our patient cohort, we observed molecular changes related to mitophagy in these accessible PBMCs, notably a significant increase in TUFM expression. Concurrently, we detected decreased vitamin D levels and increased mtDNA release in the serum of RSV-infected patients, indicative of systemic mitochondrial damage. To directly elucidate the pathogenic mechanism of RSV in its natural target cells, we utilized the human bronchial epithelial cell line BEAS-2B. This system directly models the primary site of RSV infection and injury in the human airway. It is noteworthy that the NS1-TUFM regulatory axis elucidated in BEAS-2B cells resonates with the mitophagic signatures observed in patient PBMCs. This consistency across models not only strengthens the biological reliability of our findings but also suggests that the core mechanism uncovered in bronchial epithelium, namely, RSV-driven mitophagy via the NS1-TUFM axis, likely has manifestations at the systemic immunometabolic level. Furthermore, both models collectively underscore the crucial protective role of Vitamin D within this pathway.

We acknowledge certain limitations in our study. Firstly, our reliance on NS1 plasmid transfection, while necessary for detailed mechanistic dissection under accessible biosafety conditions, cannot fully replicate the complex dynamics of live RSV infection. Secondly, the use of the Beas-2B cell line, though a relevant and standard model for human bronchial epithelium, warrants future validation in primary human airway cells and in vivo models to confirm translational relevance. Future studies should examine vitamin D’s impact on other RSV proteins (e.g., NS2, phosphoprotein, and nucleocapsid protein [Van Royen et al., 2022]) and validate findings in animal models. Additionally, the precise mechanism whereby vitamin D reduces mitochondrial TUFM, whether through transcriptional regulation, protein degradation, or impaired mitochondrial import, requires further investigation. Whether other mitochondrial proteins are also targets of NS1 is the focus of our further research.

Despite these limitations, our findings have significant translational implications. They provide a mechanistic basis for clinical observations linking vitamin D status to RSV outcomes and suggest that vitamin D supplementation could be optimized to disrupt viral hijacking of mitochondrial mass control. The demonstration that TUFM dictates both NS1 localization and mitophagy activation positions it as a compelling target for novel host-directed antivirals. Future studies should explore whether vitamin D analogs or TUFM inhibitors can therapeutically modulate this pathway in established infection.

In conclusion, this study demonstrates through a series of in vitro experiments that vitamin D inhibits mitophagy in bronchial epithelial cells infected with RSV by disrupting the NS1-TUFM interaction. Vitamin D facilitates the translocation of NS1 from mitochondria to cytosol and reduces mitochondrial TUFM accumulation, restoring mitochondrial integrity and attenuating inflammation. Crucially, TUFM overexpression abolishes these protective effects, confirming TUFM as the key mediator. These findings reveal a novel mechanism linking nutritional status (vitamin D) to mitochondrial mass control during viral infection, suggesting that the vitamin D-TUFM axis may serve as a potential therapeutic target for mitigating RSV-induced lung damage.

## Figures and Tables

**Table 1.** The information on clinical samples

	Normal	RSV-positive	*P* value
Number of subjects	10	13	/
Age (years)	3.33 ± 2.92	1.69 ± 2.03	0.1277
Male/Female (%)	40%/60%	77%/23%	0.0778
Respiratory rate (breaths per min)	27.20 ± 0.12	39.85 ± 13.76	0.0120
RSV positive (nucleic acid/antigen)	/	9/4	/
WBC count (× 10^9^)	7.50 ± 1.66	9.97 ± 3.96	0.0795
Neutrophil percentage (%)	37.17 ± 10.85	39.83 ± 24.79	0.8062
Lymphocyte count (× 10^9^)	3.82 ± 1.42	4.08 ± 1.62	0.6858
C-reactive protein (mg/L)	1.98 ± 1.71	14.17 ± 18.22	0.0496
LDHA (U/L)	196.26 ± 33.34	399.86 ± 102.53	0.0004

Note: WBC, white blood cell; LDHA, lactate dehydrogenase

**Table 2.** The sequence of PCR primers

Gene	Sequence (5’–3’)	Length (bp)
*mt-Co2*	F: GCTGTCCCCACATTAGGCTT	139
	R: CGATGGGCATGAAACTGTGG	
*n-GAPDH*	F: GGCTCCCACCTTTCTCATCC	138
	R: CTCCCCACATCACCCCTCTA	
*LC3*	F: GCCCTCAGACCGGCCTTTCA	128
	R: GCTGCTTCTCACCCTTGTAG	
*ATG5*	F: AAAGATGTGCTTCGAGATGTGT	144
	R: CACTTTGTCAGTTACCAACGTCA	
*TOMM20*	F: AAAAGAGTGGAGCAGGTGGG	102
	R: GATGCCACCAAGTACCAAGGA	
*COXIV*	F: TGGCCCATGTCAAGCACCT	172
	R: CCGCCCACAACCGTCTTCC	
*TUFM*	F: CACTTACATCCCAGTGCCCG	96
	R: GTCACCACGGTGCCACG	
*GAPDH*	F: GGTCATGAGTCCTTCCACGAT	104
	R: GGTCATGAGTCCTTCCACGAT	

**Table 3.** The information on antibodies

Name	Item number	Dilution rate	Molecular weight	Company
LC3	14600-1-AP	1: 2000	14-18 kDa	Proteintech, China
ATG5	ab108327	1: 10000	50 kDa	Abcam, USA
TOMM20	66777-1-Ig	1: 1000	16 kDa	Proteintech, China
COXIV	ab33985	1: 1000	15 kDa	Abcam, USA
RSV-NS1	MA5-50511	1: 1000	20 kDa	Thermo Fisher, USA
TUFM	ab173300	1: 20000	43 kDa	Abcam, USA
VDAC1	AWA06016	1: 1000	31 kDa	Abiowell, China
GAPDH	AWA80007	1: 5000	37 kDa	Abiowell, China

## Supplementary Information

Fig. S1. Statistics of relative gray values from Western blot analysis of human PBMCs and Beas-2B cells under various treatments. (A) Western blot assessment of relative expression levels of LC3, TUFM, ATG5, TOMM20, and COXIV in PBMCs from healthy volunteers and RSV-infected patients. n= 9. ^*^*P* < 0.05 vs. Normal group. (B) Western blot evaluation of relative expression levels of ATG5, LC3II/I, TOM20, VDAC1, and COXIV in Beas-2B cells after RSV-NS1 overexpression. n = 3. ^*^*P* < 0.05 vs. oe-NC group. (C) Western blot analysis of TUFM relative expression levels in Beas-2B cells transfected with TUFM silencing plasmids (#1–3) and control plasmid. n = 3. ^*^*P* < 0.05 vs. si-NC group. (D) Western blot analysis of relative expression levels of ATG5, LC3II/I, TOM20, VDAC1, and COXIV in Beas-2B cells overexpressing RSV-NS1 after TUFM silencing. n = 3. ^*^*P* < 0.05 vs. oe-NC group; ^#^*P* < 0.05 vs. oe-RSV-NS1+si-NC group. (E) Western blot analysis of LC3II/I and TOM20 expression changes in Beas-2B cells following vitamin D intervention at different concentrations. (F) Western blot analysis of relative expression levels of ATG5, LC3II/I, TOM20, VDAC1, and COXIV in Beas-2B cells treated with 10 μM vitamin D. n = 3. ^*^*P* < 0.05 vs. oe-NC group; ^#^*P* < 0.05 vs. oe-RSV-NS1 group. (G) Western blot analysis of relative expression levels of ATG5, LC3II/I, TOM20, VDAC1, and COXIV in Beas-2B cells overexpressing TUFM after 10 μM vitamin D intervention. n = 3. ^*^*P* < 0.05 vs. oe-RSV-NS1 group; ^#^*P* < 0.05 vs. oe-RSV-NS1+VitD+oe-NC group.

Fig. S2. Sorting and validation of NS1-positive Beas-2B cells. (A) Specific sorting of NS1-positive (NS1^+^) cell populations based on fluorescence signals using flow cytometry. (B) Immunofluorescence staining validation of NS1-positive cells. Scale bar = 50 μm.

Fig. S3. Verification of the purity of cellular fractions following separation of cytosolic and mitochondrial components by differential velocity centrifugation. Western blot analysis of GADPH and COX-IV signals in the cytosolic fraction and mitochondrial fraction.

Fig. S4. Autophagy inhibitor Bafilomycin A1 intervention rescues mitophagy induced by RSV-NS1 overexpression. Beas-2B cells were pretreated with 10 μM Bafilomycin A1 for 2 h. Immunofluorescence colocalization analysis of LC3 and TOMM20 to assess mitophagy. Scale bar = 25 μm. n = 3. ^*^*P* < 0.05 vs. oe-NC group; ^#^*P* < 0.05 vs. oe-RSV-NS1 group.
