# Supplementary material for: Vitamin D disrupts NS1-TUFM interaction to suppress pathogenic mitophagy in RSV-induced mitochondrial injury of bronchial epithelial cells
Source: J Microbiol. 2026 Jan 31;64(1):e2508009. doi: 10.71150/jm.2508009 (PMC13577116; doi:10.71150/jm.2508009)
Supplement: Supplementary file 1 [file jm-2508009-Supplementary-Fig-S1.pdf]

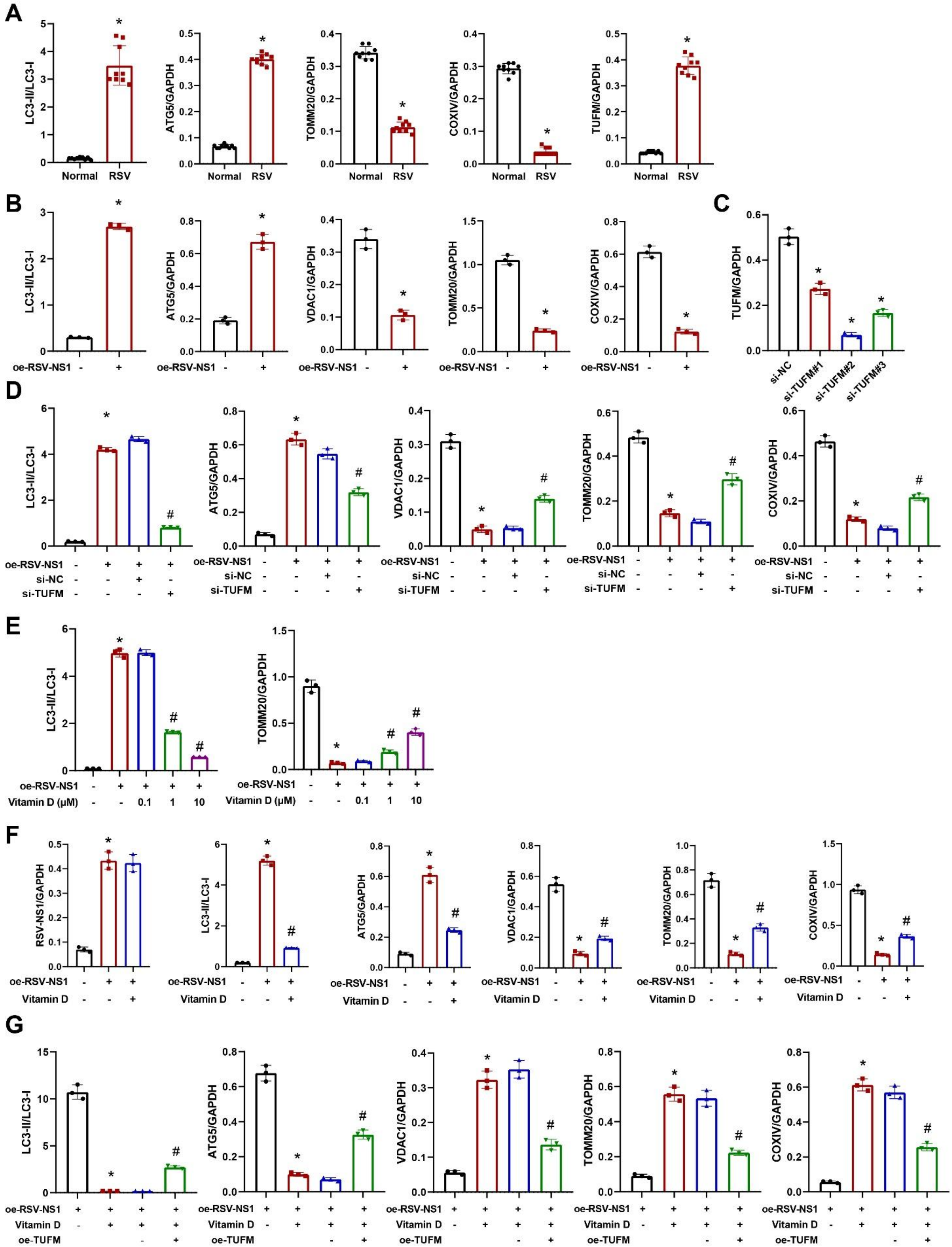

Fig. S1. Statistics of relative gray values from Western blot analysis of human PBMCs and Beas-

2B cells under various treatments. (A) Western blot assessment of relative expression levels of LC3, TUFM, ATG5, TOMM20, and COXIV in PBMCs from healthy volunteers and RSV-infected patients. n= 9. $^{*}P < 0.05$ vs. Normal group. (B) Western blot evaluation of relative expression levels of ATG5, LC3II/I, TOM20, VDAC1, and COXIV in Beas-2B cells after RSV-NS1 overexpression. n = 3. $^{*}P < 0.05$ vs. oe-NC group. (C) Western blot analysis of TUFM relative expression levels in Beas-2B cells transfected with TUFM silencing plasmids (#1–3) and control plasmid. n = 3. $^{*}P < 0.05$ vs. si-NC group. (D) Western blot analysis of relative expression levels of ATG5, LC3II/I, TOM20, VDAC1, and COXIV in Beas-2B cells overexpressing RSV-NS1 after TUFM silencing. n = 3. $^{*}P < 0.05$ vs. oe-NC group; $^{\#}P < 0.05$ vs. oe-RSV-NS1+si-NC group. (E) Western blot analysis of LC3II/I and TOM20 expression changes in Beas-2B cells following vitamin D intervention at different concentrations. (F) Western blot analysis of relative expression levels of ATG5, LC3II/I, TOM20, VDAC1, and COXIV in Beas-2B cells treated with 10 μM vitamin D. n = 3. $^{*}P < 0.05$ vs. oe-NC group; $^{\#}P < 0.05$ vs. oe-RSV-NS1 group. (G) Western blot analysis of relative expression levels of ATG5, LC3II/I, TOM20, VDAC1, and COXIV in Beas-2B cells overexpressing TUFM after 10 μM vitamin D intervention. n = 3. $^{*}P < 0.05$ vs. oe-RSV-NS1 group; $^{\#}P < 0.05$ vs. oe-RSV-NS1+VitD+oe-NC group.
