# Supplementary material for: Vitamin D disrupts NS1-TUFM interaction to suppress pathogenic mitophagy in RSV-induced mitochondrial injury of bronchial epithelial cells
Source: J Microbiol. 2026 Jan 31;64(1):e2508009. doi: 10.71150/jm.2508009 (PMC13577116; doi:10.71150/jm.2508009)
Supplement: Supplementary file 2 [file jm-2508009-Supplementary-Fig-S2.pdf]

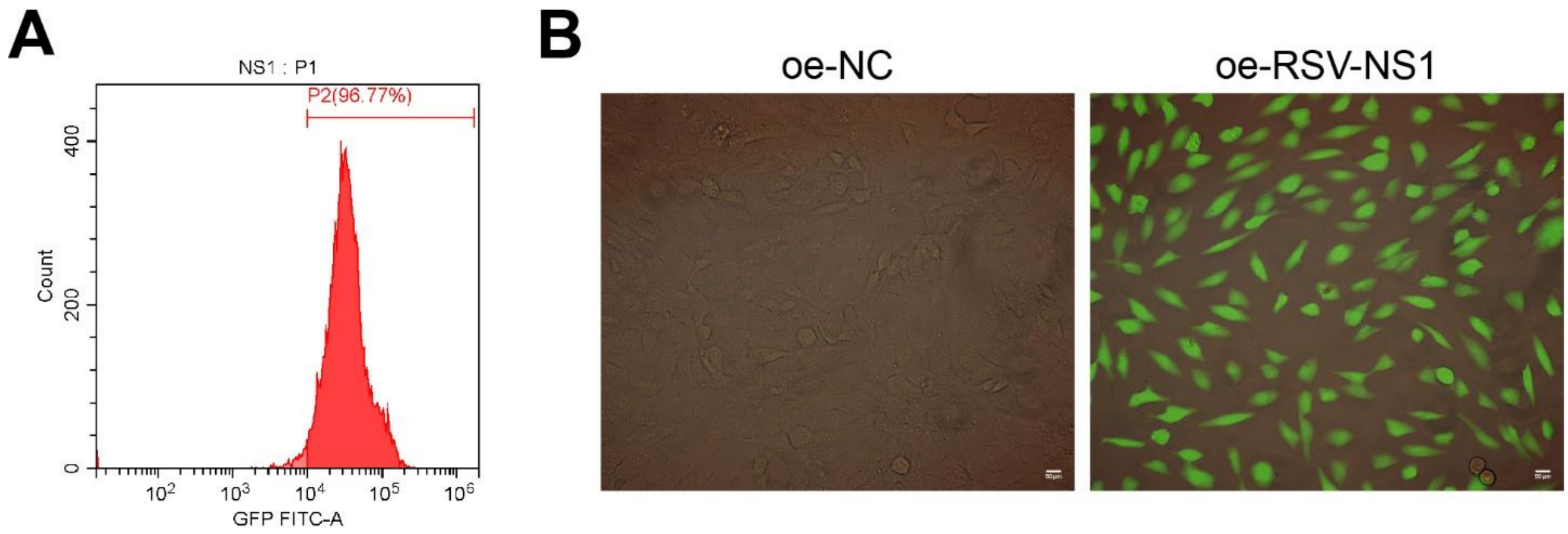

Fig. S2. Sorting and validation of NS1-positive Beas-2B cells. (A) Specific sorting of NS1-positive ($NS1^+$) cell populations based on fluorescence signals using flow cytometry. (B) Immunofluorescence staining validation of NS1-positive cells. Scale bar = 50 μm.
