# Supplementary material for: Vitamin D disrupts NS1-TUFM interaction to suppress pathogenic mitophagy in RSV-induced mitochondrial injury of bronchial epithelial cells
Source: J Microbiol. 2026 Jan 31;64(1):e2508009. doi: 10.71150/jm.2508009 (PMC13577116; doi:10.71150/jm.2508009)
Supplement: Supplementary file 3 [file jm-2508009-Supplementary-Fig-S3.pdf]

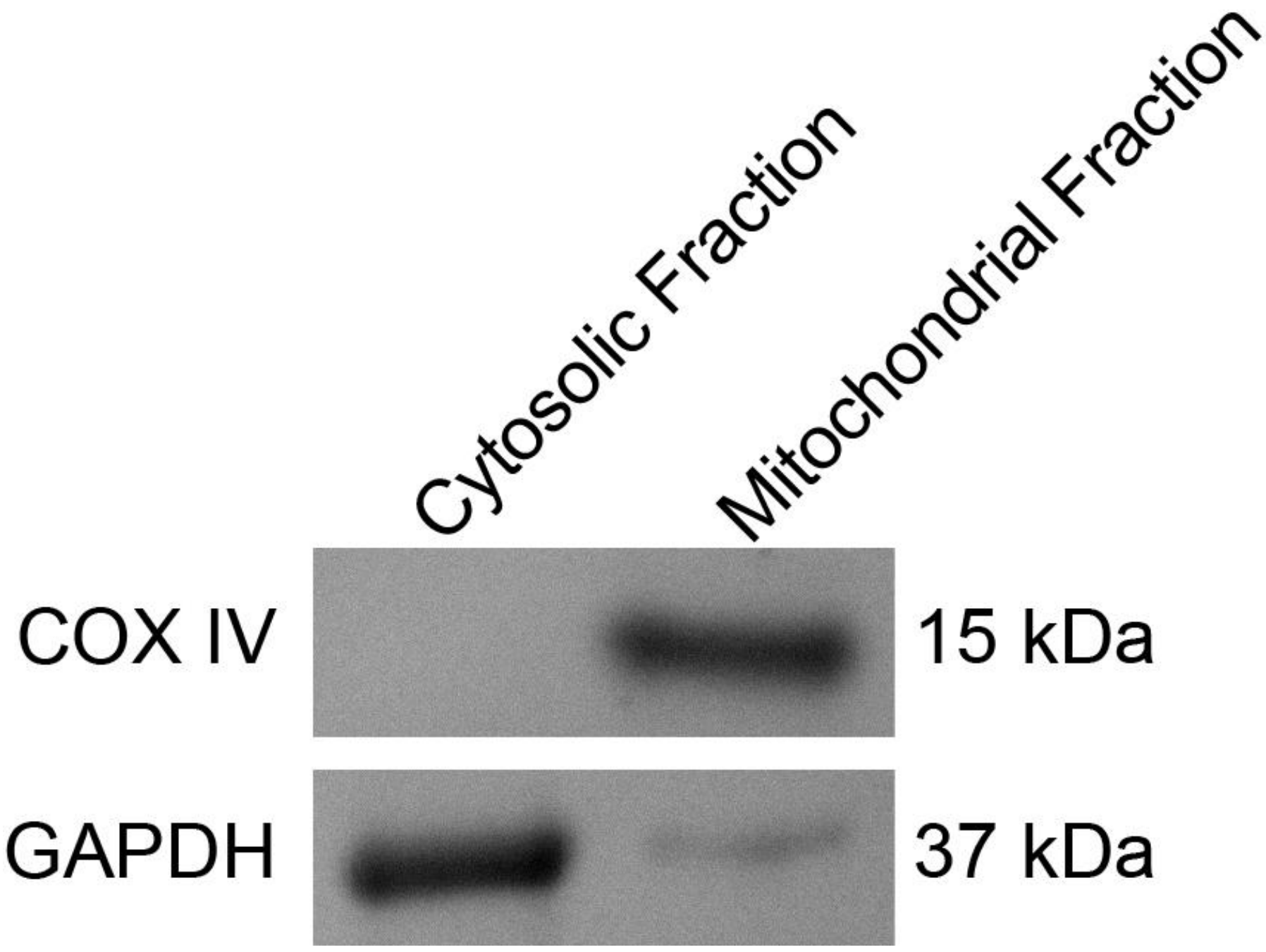

Fig. S3. Verification of the purity of cellular fractions following separation of cytosolic and mitochondrial components by differential velocity centrifugation. Western blot analysis of GADPH and COX-IV signals in the cytosolic fraction and mitochondrial fraction.
