# Supplementary material for: Vitamin D disrupts NS1-TUFM interaction to suppress pathogenic mitophagy in RSV-induced mitochondrial injury of bronchial epithelial cells
Source: J Microbiol. 2026 Jan 31;64(1):e2508009. doi: 10.71150/jm.2508009 (PMC13577116; doi:10.71150/jm.2508009)
Supplement: Supplementary file 4 [file jm-2508009-Supplementary-Fig-S4.pdf]

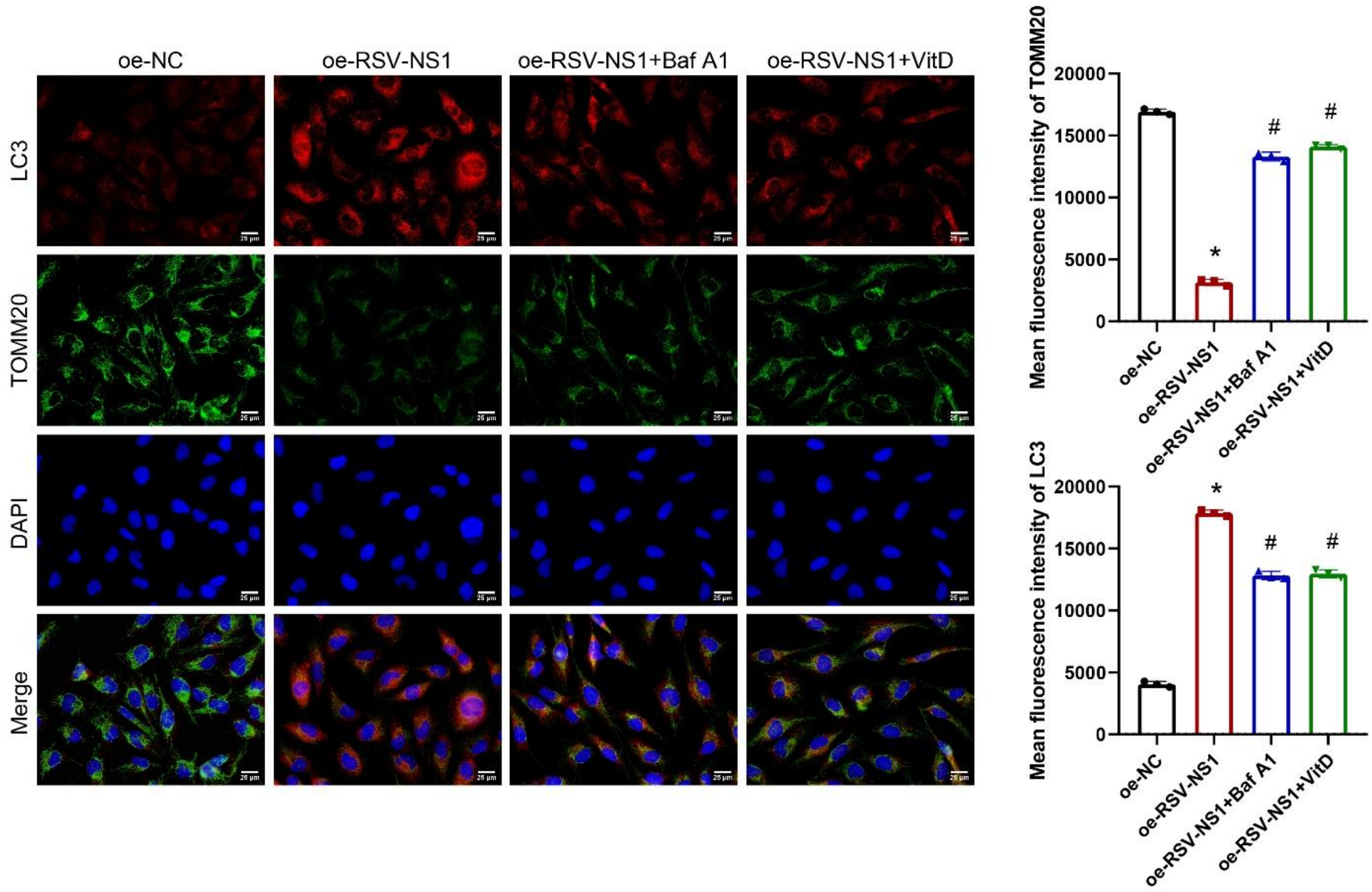

Fig. S4. Autophagy inhibitor Bafilomycin A1 intervention rescues mitophagy induced by RSV-NS1 overexpression. Beas-2B cells were pretreated with 10 μM Bafilomycin A1 for 2 h. Immunofluorescence colocalization analysis of LC3 and TOMM20 to assess mitophagy. Scale bar = 25 μm. n = 3. $^{*}P < 0.05$ vs. oe-NC group; $^{\#}P < 0.05$ vs. oe-RSV-NS1 group.
